# Evaluation of Femoral Bone Fracture Healing in Rats by the Modal Damping Factor and Its Correlation With Peripheral Quantitative Computed Tomography

**DOI:** 10.7759/cureus.13342

**Published:** 2021-02-15

**Authors:** Stavros Chalikias, Nikolaos Papaioannou, George Koundis, Eleni Pappa, Antonios Galanos, George Anastassopoulos, Ioannis N Sarris, Sofia Panteliou, Efstathios Chronopoulos, Ismene A Dontas

**Affiliations:** 1 Department of Orthopedics, Golden Jubilee National Hospital, Glasgow, GBR; 2 Laboratory for Research of the Musculoskeletal System, KAT General Hospital, School of Medicine, National and Kapodistrian University of Athens, Athens, GRC; 3 5th Department of Orthopedics, KAT General Hospital, Athens, GRC; 4 Department of Mechanical Engineering and Aeronautics, University of Patras, Patras, GRC; 5 2nd Department of Orthopedics, Konstantopouleio General Hospital, School of Medicine, National and Kapodistrian University of Athens, Athens, GRC

**Keywords:** bone mineral density, femur, fracture healing, modal damping factor, peripheral quantitative computed tomography, rat, strength strain index

## Abstract

Introduction

Monitoring the progress of fracture healing is essential in order to establish the appropriate timing that ensures adequate bone strength for weight-bearing. In the present experimental study on a rat model of femoral fracture healing, the measurement of bone density and strength by peripheral quantitative computerized tomography (pQCT) was correlated with the modal damping factor (MDF) method.

Methods

Four groups of 12 male six-month-old Wistar rats each were anesthetized and submitted to baseline femoral pQCT and MDF scanning, followed by aseptic midshaft osteotomy of the right femur which was fixed by a locking intramedullary nail technique. The animals were left to recover and re-scanned following euthanasia of each group after six, eight, 10, and 12 weeks, respectively. The parameters measured by the pQCT method were total bone mineral density (BMD) and polar strength strain index (SSIp).

Results

Fracture healing progressed over time and at 12 weeks post-osteotomy there was no statistically significant difference between the osteotomized right and the control left femurs regarding MDF, BMD, and SSIp measurements. The highest correlations for the osteotomized femurs were observed between MDF and BMD (r = -0.647, P = 0.043), and between MDF and SSIp (r = -0.350, P = 0.321), at 10 weeks postoperatively. The high to moderate correlations between MDF and BMD, and between MDF and SSIp respectively, support the validity of MDF in assessing fracture healing.

Conclusions

Based on our findings in this fracture healing animal model, the results from the MDF method are reliable and correlate highly with the total BMD and moderately with the SSI polar values obtained by the pQCT method of bone quality measurement. Further studies are needed which may additionally support that the MDF method can be an attractive portable alternative to monitor fracture healing in the community.

## Introduction

Monitoring of the progress of fracture healing is an open scientific question concerning objective measurements that determine each phase of bone healing in order to clarify its sufficient mechanical strength. The fracture healing process consists of different phases, well described in the literature [1,2]. Τhe initial injury generates a hematoma and triggers an acute inflammatory response which is the first basic stage. This leads to soft callus formation, later transformed into a hard callus. The remodeling phase follows and culminates in producing the same bone tissue as the initial normal bone. To date, usual methods of evaluation of bone healing are mainly radiography, CT scans, and, optionally, magnetic resonance imaging [3,4]. Additionally, more refined but not commonly used methods in clinical practice are peripheral quantitative computed tomography (pQCT) and high-resolution peripheral quantitative computed tomography (HRpQCT) [5,6]. For experimental purposes only, micro-CT, Raman Spectroscopy, and the three-point-bending *ex vivo* test are also used [3,7].

The Modal Damping Factor (MDF) is a non-invasive method that is based on the assessment of bone dynamic characteristics by applying vibration excitation in the range of acoustic frequencies, in the form of an acoustic sweep signal. As structural quality deteriorates, the MDF value increases and vice versa [8]. The method has already been successfully applied on metallic structures and composites, as well as on bones, and is supported by analytical and arithmetic tools based on the model's theory [8].

According to our previous research findings, MDF is a user-friendly, non-expensive method, and has been proven to relate directly to bone strength and account for its structural changes [9-13]. The present study aimed to investigate whether MDF could serve as an additional objective, a quantitative and competitive tool for monitoring the process of bone fracture healing in rats’ femora, and compare the findings with results obtained from pQCT. The pQCT method is the only available method that can provide, in addition to bone quality parameters such as bone mineral density, mechanical strength data through the polar strength strain index (SSIp) [14].

## Materials and methods

Animals

For the present study, 48 six-month-old male Wistar rats were used (source of purchase: Institut Pasteur Hellenique, Athens, Greece). All animal procedures were performed according to the Greek Presidential Decree 56/13 'for the protection of animals used for experimental purposes' which conforms to the Directive 2010/63/EU. The study design was approved by the Institution's Protocol Evaluation Committee and licensed by the Directorate of Veterinary Services, Prefecture of Attica, Greece (permit no. 3003/15.05.2014). The study took place in the Laboratory for Research of the Musculoskeletal System, School of Medicine, National & Kapodistrian University of Athens, KAT Hospital (user establishment license no. EL 25 BIO 018). The rats were housed two to a cage, with temperature 21±2°C, 50% relative humidity, lighting conditions 12/12 hours light/dark, administered standard rat chow (proteins 27%) and bottled tap water *ad libitum*.

The rats used in this study were divided into four groups, each of them containing 12 subjects, which was determined following sample size estimation. The groups were named A, B, C, and D according to their time of euthanasia (6, 8, 10, and 12 weeks postoperatively respectively). Littermates were randomized into each of the four groups.

Pilot experiment with and without soft tissues

Prior to the main fracture healing experiment, a pilot experiment was conducted on two rats euthanized for a different protocol, to investigate the effect that soft tissues may have on the parameters to be assessed. The midshaft of both the right and left femurs of the animals was shaved and measured by MDF. The soft tissues were then removed and the midshaft of the femurs was re-measured. There was no difference in the parameters measured with and without soft tissues.

Osteotomy surgery 

For the femoral midshaft osteotomy, the rats were anesthetized by i.m. injection of ketamine hydrochloride (75 mg/kg body weight) and dexmedetomidine (0.5 mg/kg), given pre-emptive analgesia by s.c. injection of carprofen (4 mg/kg) and chemoprophylaxis by s.c. injection of enrofloxacin (10 mg/kg) prior to surgery as well as on postoperative days one and two. Anesthesia was reversed by i.m. administration of atipamezole (0.02 ml/100 g). The rats were additionally subjected to ketamine/dexmedetomidine anesthesia with atipamezole reversal in order to be briefly immobilized for pre-operative pQCT and MDF measurements one week prior to surgery.

At day zero, by aseptic technique, the knee joint was exposed through a midline skin incision and medial parapatellar approach by two orthopedic surgeons blinded to the group assignments. Access to the medullary cavity was achieved via the intercondylar entry point and the medullary cavity was prepared with a 1.65 mm reamer to accept the "RatNail" of the "Retrograde intramedullary locking nailing system" (RISystem AG, Switzerland) [15,16]. A second lateral incision and blunt dissection of the vastus lateralis were performed to expose the middle shaft of the right femur. Two locking pins were applied via the aiming device on each side of the planned osteotomy site, achieving interlocking of the nail. Using the special saw guide of the system, a defined osteotomy was performed at the femoral midshaft between the two locking pins using a 0.44 mm Gigli wire saw, under irrigation with normal saline solution to minimize thermal injury. The wounds were closed in layers.

All animals recovered from surgery uneventfully and were allowed unrestricted full weight-bearing immediately postoperatively. The fracture remained fixed by the intramedullary RatNail throughout the study period. Following the femoral osteotomy, each respective group containing 12 rats were euthanized at postoperative weeks six, eight, 10, and 12 of the healing process. According to the literature, fracture union (callus) is achieved at 12 weeks or even later in adult rats [17,18]. Therefore, we chose to monitor the evolution of the fracture healing process in conjunction with pQCT and MDF for up to 12 weeks.

Tissue collection and parameters measured

Photographs

Immediately after euthanasia, both the osteotomized and non-osteotomized femur of each rat were carefully disarticulated, excised, and the surrounding soft tissues removed, without affecting the fracture callus. All femurs were photographed.

Radiographs

Immediately after euthanasia, the right femurs were X-rayed, obtaining anterioposterior (A/P) and lateral views, from a distance of 30 cm, with elements of 45 kV and 2.20 mAs (SIEMENS AXIOM Sireskop SD33, ISO certified), both with the intramedullary nail and without it after its careful removal.

pQCT

Measurements of both the right and left femurs of all rats were conducted by a pQCT scanner (Stratec XCT 2000, Stratec Medizintechnik GmbH, Pforzheim, Germany, software version 6.20). Its repeatability was 0.2 % with the phantom and 1% with *in vitro* measurements. Scanning was carried out twice; once one week before osteotomy surgery *in vivo* under anesthesia and once after euthanasia *ex vivo*. After an initial scout view of the femur, the osteotomy site at the midshaft of the right (osteotomized) femurs and the left (control) femurs were scanned. The *in vivo* pQCT measurement with soft tissues of each femur was compared with the *ex vivo* measurement without soft tissues; the *ex vivo* measurement of the right femur was conducted after removal of the intramedullary nail. The parameters measured were total bone mineral density (BMD) in mg/cm^3^ and polar strength strain index (SSIp) in mm^3^.

MDF

Measurements of both right and left femurs of all rats were carried out twice by MDF; once one week before osteotomy surgery *in vivo* under anesthesia and once after euthanasia *ex vivo*. MDF is a dimensionless number, with values in the interval (0, 1), with higher values corresponding to deteriorated structure quality and vice versa [8]. Femoral callus quality was assessed with MDF, thus obtaining two groups of data, corresponding to bones with and without soft tissues. As occurred with the pQCT measurements, the MDF initial measurement with soft tissues of each femur was compared with the *ex vivo* measurement without soft tissues; the *ex vivo* measurement of the right femur was conducted after removal of the intramedullary nail.

Statistical analysis

Data are expressed as mean±standard deviation (SD) for continuous variables and as percentages for categorical data. The Kolmogorov-Smirnov test was utilized for normality analysis of the parameters. The construct validity is used to determine how accurately a test measures what it is supposed to measure and is verified by comparing the same to other tests that measure similar qualities evaluating the degree of correlation between the two. Construct validity of MDF was determined by establishing its correlation to the variables total BMD and SSIp using the Pearson’s correlation coefficient. A moderate or high correlation between MDF to the well-established measures BMD and SSIp would support the validity of MDF in measuring fracture healing.

Comparisons of variables during the observation period (baseline vs time of euthanasia) were performed using paired-samples t-test or Wilcoxon test in case of violation of normality. A one-way analysis of variance (ANOVA) model was used for the comparison of different time measurements of variables (six weeks vs eight weeks vs 10 weeks vs 12 weeks). Pair-wise multiple comparisons were performed using the method of the Bonferroni test. Kruskal-Wallis and Mann-Whitney tests were used in case of violation of normality. The comparisons of variables between the osteotomized right and control left femur of each animal in every group were examined using paired samples t-test or Wilcoxon test in case of violation of normality.

All tests were two-sided, statistical significance was set at P < 0.05. All analyses were carried out using the Statistical Package for the Social Sciences (SPSS) version 21.00 (IBM Corp., Somers, NY).

## Results

**Figure 1:**
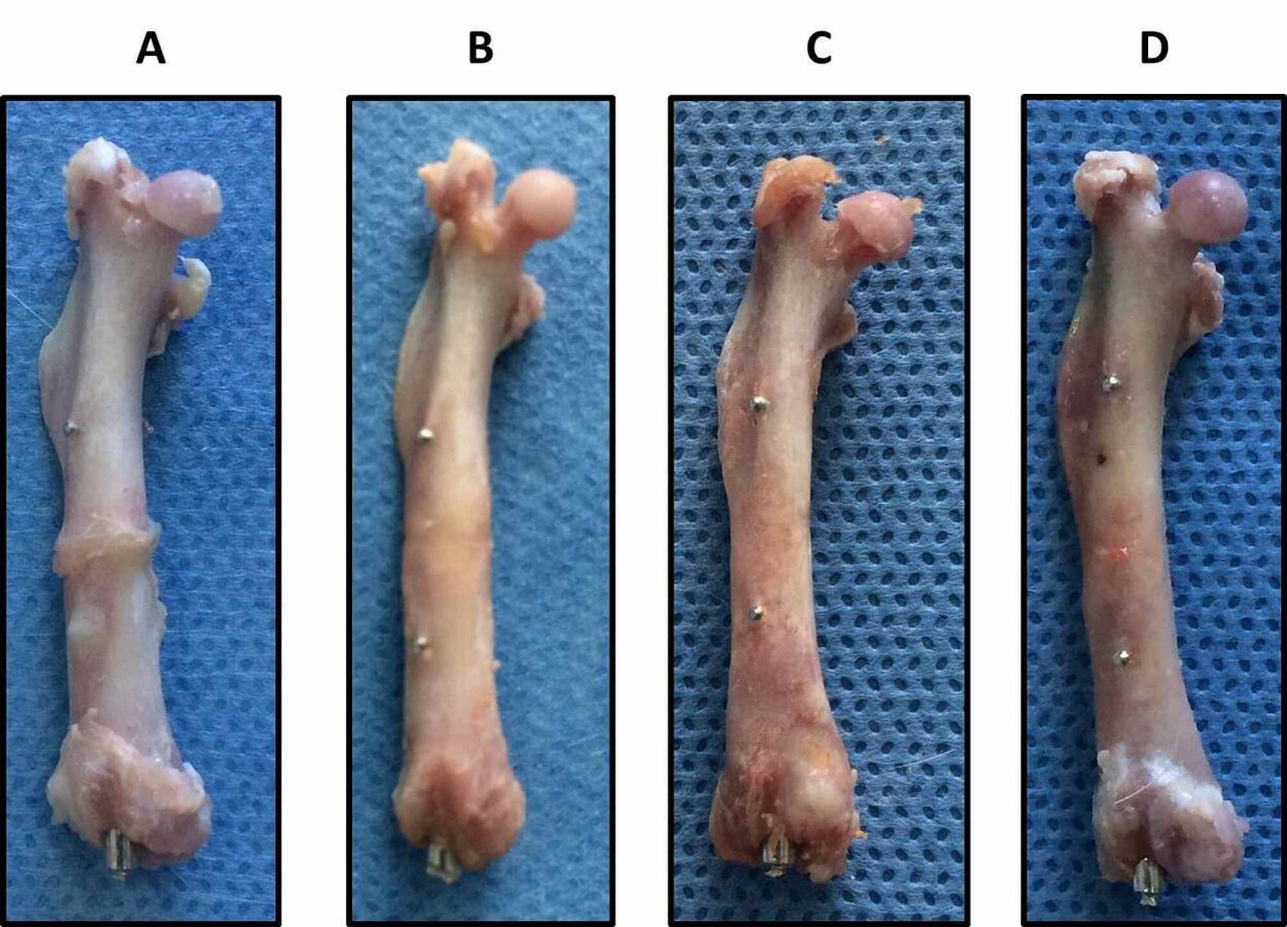
Photographs of right (osteotomized) femurs of rats from groups A, B, C, and D at the respective euthanasia times (six, eight, 10, and 12 weeks postoperatively) with the locked intramedullary nail in place.

**Figure 2:**
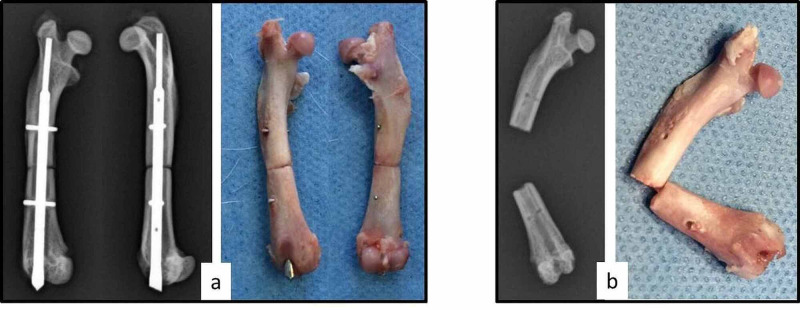
Anterioposterior and lateral radiographs and respective photographs of a failed healing femur of one rat (a) before and (b) after removal of the intramedullary nail.

Photographs of indicative right (osteotomized) femurs of rats from groups A, B, C, and D following their disarticulation and removal of soft tissues are shown in Figure 1, where the process of fracture healing through time can be seen. The still enlarged callus at six postoperative weeks can be seen in Figure 1A, visible remnants of the osteotomy are seen in Figure 1B, whereas in Figures 1C, 1D, the osteotomy can barely be seen. All femurs of each group presented similar healing, except for one rat in group A where the union was not achieved at the group's time of euthanasia (six weeks post-osteotomy). Although up until euthanasia the rat clinically appeared to have a normally healing femur, after euthanasia, disarticulation, and removal of soft tissues, the absence of callus was observed, while following removal of the intramedullary nail, the femur's non-union became evident (Figures 2a, 2b). This rat's preoperative BMD, SSIp and MDF measurements were within average, however, it was replaced by an additional animal.

**Figure 3:**
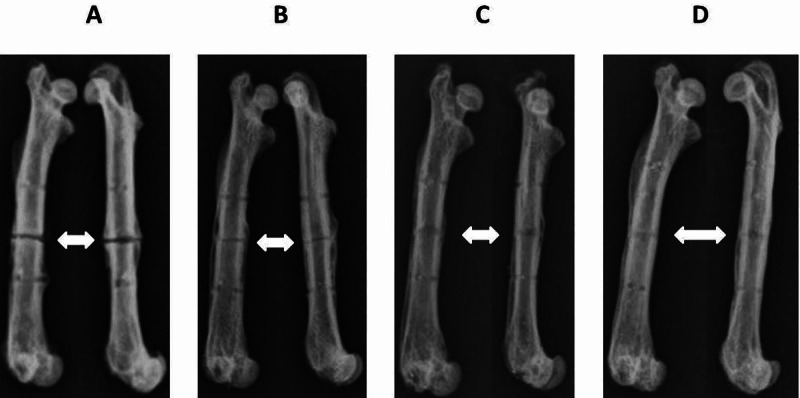
Radiographs of right (osteotomized) femurs in the anterioposterior and lateral views, of rats from groups A, B, C, and D at the respective euthanasia times (six, eight, 10, and 12 weeks postoperatively). The white arrows indicate the osteotomy site on the femoral midshaft.

Radiographs of indicative right (osteotomized) femurs of rats from groups A, B, C, and D following their disarticulation, removal of the soft tissues, and the intramedullary nail are shown in Figure 3, where the process of fracture healing through time can be seen. Anterioposterior and lateral views of the same femur are shown. The osteotomy gap at six postoperative weeks is evident in Figure 3A, as well as the callus thickening around the osteotomy site. The osteotomy gap and the callus thickening are less evident in Figure 3B. In Figures 3C, 3D, the gap is even less evident and the callus thickening has been absorbed.

pQCT

**Figure 4:**
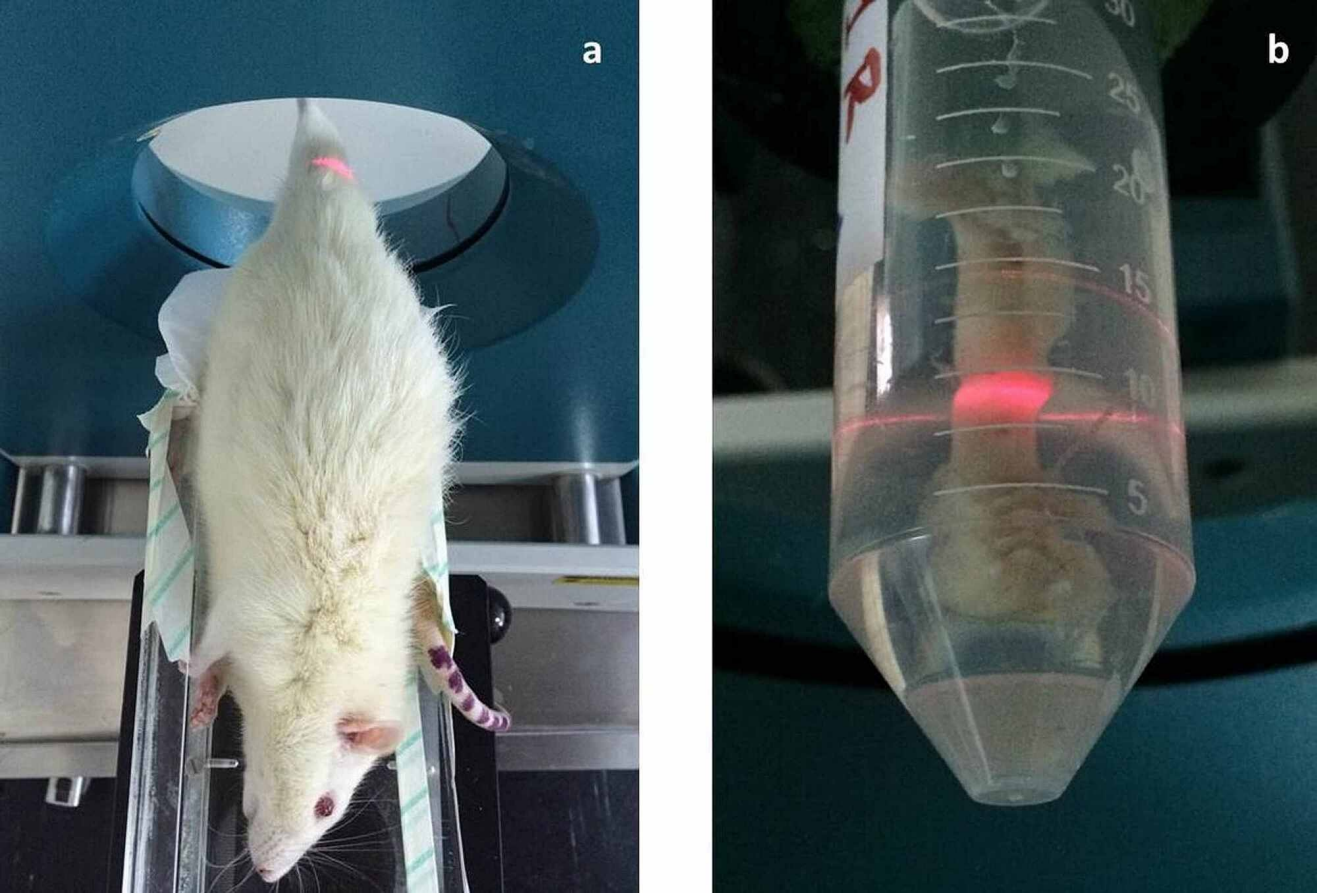
Scanning of the right femur by pQCT. Photographs of the pQCT scanning set up* in vivo* (a) and of the harvested right femur following euthanasia and removal of the soft tissues *ex vivo* (b). pQCT: peripheral quantitative computerized tomography

The femurs were scanned preoperatively under anaesthesia and postoperatively after euthanasia (Figures 4a, 4b, respectively) and the results were compared within each group. Total bone mineral density results assessed by pQCT at the midshaft of the left (control) femur and at the osteotomy site (midshaft) of the right femur of each group (time point) are provided in Table 1. All preoperative values of total BMD were similar in both left and right femurs, to begin with. There was a statistically significant increase in the total BMD values of the left (control) femurs postoperatively in all groups. The total BMD values of the right (osteotomized) femurs were increased at all euthanasia time points compared to baseline values, however, there was a statistically significant increase only at the sixth postoperative week time point (P = 0.007). The SSIp of the femoral midshaft was provided by the pQCT scan according to the innate calculation of the software.

**Table 1:** Baseline and euthanasia total BMD, MDF, and SSIp mean values ± SD of both left (L, control) and right (R, osteotomized) femurs for each group (euthanasia time point) of fracture healing. Total BMD values are in mg/cm^3^; SSIp values are in mm^3^. Pairwise comparisons between groups: *P < 0.05 vs eight weeks, **P < 0.005 vs eight weeks, ^†^P< 0.05 vs six weeks, ^††^P < 0.005 vs six weeks. BMD: bone mineral density; MDF: modal damping factor; SSIp: polar strength strain index

	Time	Group A (6 weeks)	Group B (8 weeks)	Group C (10 weeks)	Group D (12 weeks)	P-value _between groups_
Total BMD control (L)	Baseline	948.68±34.31	970.58±51.84	949.76±86.78	977.93±15.30	0.083
	Euthanasia	1033.87±45.23	1067.18±36.86	1045.57±28.63	1072.48±33.49^†^	0.042
	P-value	<0.001	<0.001	0.007	<0.001	
Total BMD osteotomized (R)	Baseline	947.06±57.03	981.10±34.70	966.44±81.69	1011.58±28.00^†^	0.050
	Euthanasia	1014.65±28.76	1030.55±84.67	1033.80±69.17	1034.80±52.77	0.861
	P-value	0.007	0.069	0.070	0.157	
MDF control (L)	Baseline	0.033±0.003	0.033±0.003	0.030±0.003	0.030±0.002	0.102
	Euthanasia	0.032±0.003**	0.027±0.002	0.031±0.002**	0.033±0.003**	<0.001
	P-value	0.455	<0.001	0.758	0.053	
MDF osteotomized (R)	Baseline	0.032±0.003	0.033±0.003	0.029±0.001*	0.030±0.002*	0.010
	Euthanasia	0.054±0.008	0.046±0.009^††^	0.037±0.006^††^	0.040±0.009^††^	<0.001
	P-value	<0.001	<0.001	0.004	0.004	
SSIp control (L)	Baseline	9.93±1.55	10.54±1.02	11.32±2.73	10.47±1.07	0.491
	Euthanasia	9.22±1.10	10.05±0.93	9.88±1.54	10.86±1.29	0.076
	P-value	0.369	0.232	0.128	0.427	
SSIp osteotomized (R)	Baseline	9.63±2.24	9.81±1.40	10.32±3.39	9.77±0.90	0.950
	Euthanasia	12.06±3.05	12.41±2.37	11.31±2.37	11.43±2.08	0.108
	P-value	0.039	0.004	0.420	0.032	

MDF measurements were obtained at the midshaft of the left (control) femur and at the osteotomy site of the right femur of each group (time point) and are provided in Table 1. The femurs were evaluated preoperatively (baseline) and postoperatively (at euthanasia), and the results were compared within each group. All preoperative values of MDF were in the same range in the left and right femurs, to begin with. MDF values of the osteotomized right femurs were higher postoperatively, which corresponds to deteriorated bone structure quality.

**Figure 5:**
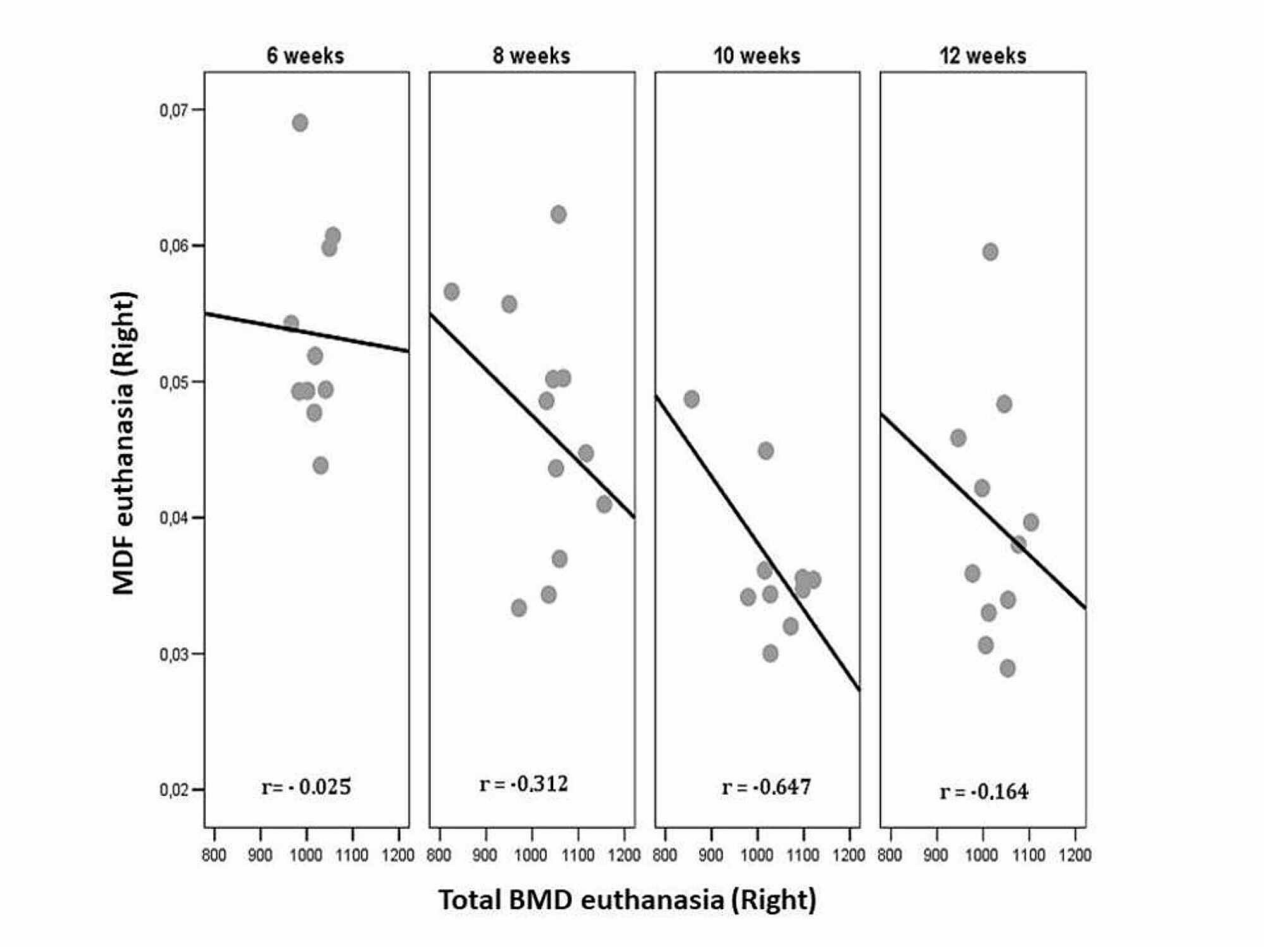
Correlation between total BMD and MDF euthanasia values of the right (osteotomized) femur for each group (euthanasia time point) of fracture healing. BMD: bone mineral density; MDF: modal damping factor

**Figure 6:**
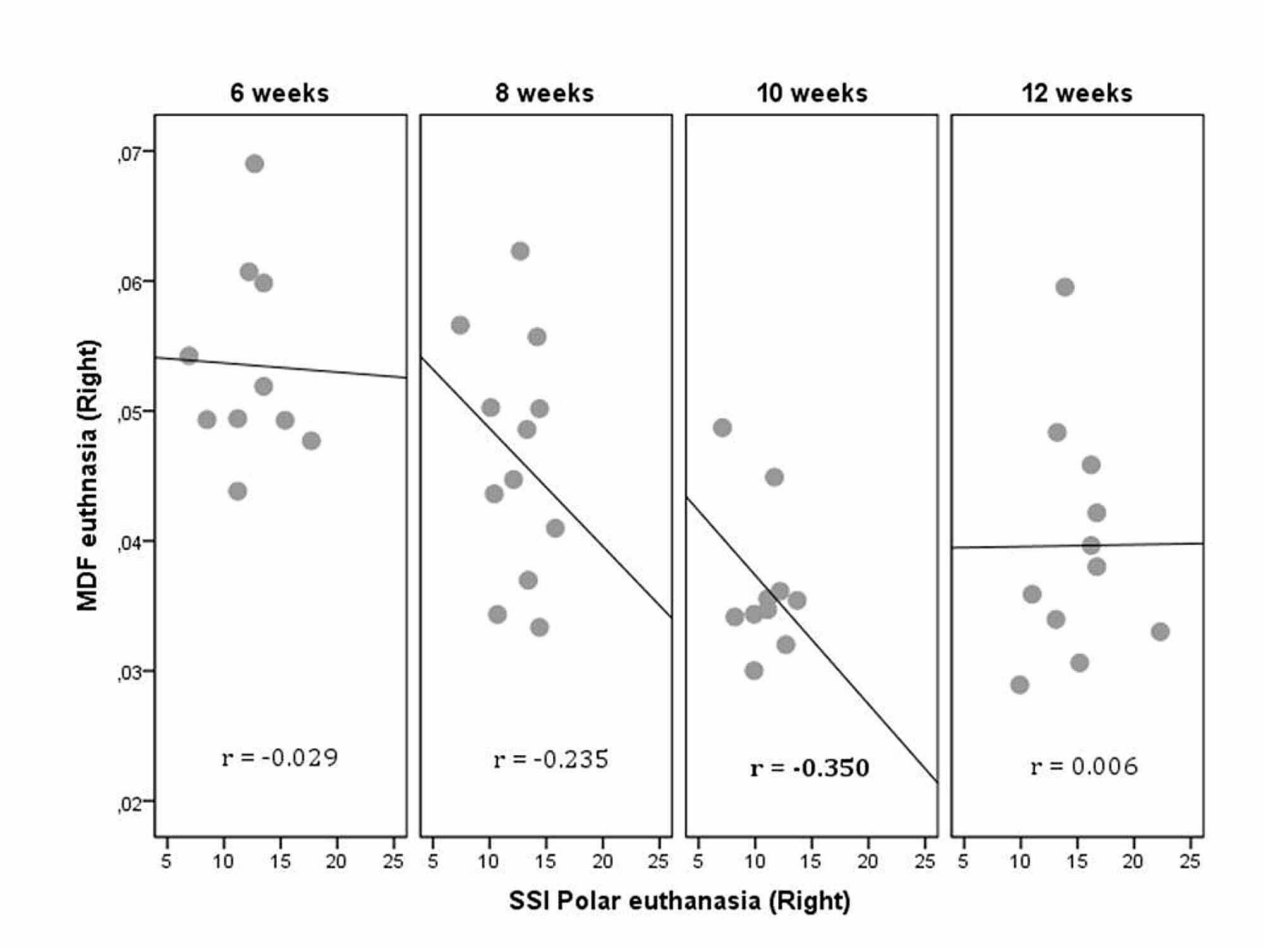
Correlation between SSIp and MDF euthanasia values of the right (osteotomized) femur for each group (euthanasia time point) of fracture healing. MDF: modal damping factor; SSIp: polar strength strain index

The euthanasia values of the three parameters evaluated, total BMD, MDF, and SSIp, of the left (control) and right (osteotomized) femur are compared separately for each group and the results are indicated in Table 2. The correlations between MDF-BMD and MDF-SSI Polar over time are shown in Tables 3, 4, and Figures 5, 6.

**Table 2:** Comparison between the left (L, control) and right (R, osteotomized) femur of total BMD, MDF, and SSIp euthanasia mean values ± SD for each group (euthanasia time point) of fracture healing. Total BMD values are in mg/cm^3^; SSIp values are in mm^3^. BMD: bone mineral density; MDF: modal damping factor; SSIp: polar strength strain index

	Femur	Group A (6 weeks)	Group B (8 weeks)	Group C (10 weeks)	Group D (12 weeks)
Total BMD euthanasia	control (L)	1033.87±45.23	1067.18±36.86	1045.57±28.63	1072.48±33.49
	osteotomized (R)	1014.65±28.76	1030.55±84.67	1033.80±69.17	1034.80±52.77
	P-value	0.282	0.139	0.601	0.077
MDF euthanasia	control (L)	0.032±0.003	0.027±0.002	0.031±0.002	0.033±0.003
	osteotomized (R)	0.054±0.008	0.046±0.009	0.037±0.006	0.040±0.009
	P-value	<0.001	<0.001	0.026	0.059
SSIp euthanasia	control (L)	9.22±1.10	10.05±0.93	9.88±1.54	10.86±1.29
	osteotomized (R)	12.06±3.05	12.41±2.37	11.31±2.37	11.43±2.08
	P-value	0.010	0.014	0.008	0.428

**Table 3:** Correlation between total BMD and MDF euthanasia values of the left (L, control) and right (R, osteotomized) femur for each group (euthanasia time point) of fracture healing. Total BMD values are in mg/cm^3^. BMD: bone mineral density; MDF: modal damping factor

Total BMD euthanasia values (L)		MDF euthanasia values (L)			
		Group A (6 weeks)	Group B (8 weeks)	Group C (10 weeks)	Group D (12 weeks)
	Pearson’s (r)	-0.424	0.361	-0.180	0.051
	P-value	0.194	0.248	0.618	0.876
Total BMD euthanasia values (R)		MDF euthanasia values (R)			
	Pearson’s (r)	-0.025	-0.312	-0.647	-0.164
	P-value	0.945	0.323	0.043	0.630
Total BMD euthanasia values ratio R/L		MDF euthanasia values ratio R/L			
	Pearson’s (r)	-0.090	-0.383	-0.662	-0.199
	P-value	0.806	0.219	0.037	0.557

**Table 4:** Correlation between SSIp and MDF euthanasia values of the left (L, control) and right (R, osteotomized) femur for each group (euthanasia time point) of fracture healing. SSIp values are in mm^3^. MDF: modal damping factor; SSIp: polar strength strain index

SSIp euthanasia values (L)		MDF euthanasia values (L)			
		Group A (6 weeks)	Group B (8 weeks)	Group C (10 weeks)	Group D (12 weeks)
	Pearson’s (r)	0.473	0.510	0.493	-0.134
	P-value	0.142	0.090	0.147	0.678
SSIp euthanasia values (R)		MDF euthanasia values (R)			
	Pearson’s (r)	-0.029	-0.235	-0.350	-0.006
	P-value	0.937	0.461	0.321	0.987
SSIp euthanasia values Ratio R/L		MDF euthanasia values ratio R/L			
	Pearson’s (r)	0.054	0.055	-0.573	-0.061
	P-value	0.881	0.864	0.054	0.858

Total BMD results

There is no statistically significant difference between the groups (six weeks vs eight weeks vs 10 weeks vs 12 weeks) of the baseline values of the control left (L) femurs (P = 0.083) and no statistically significant difference between the groups (six weeks vs eight weeks vs 10 weeks vs 12 weeks) of the euthanasia values of the osteotomized right (R) femurs (P = 0.861, Table 1).

However, there is a statistically significant difference of the euthanasia total BMD values of the control left (L) femurs (P = 0.042) (pairwise comparisons indicate a marginal difference between the sixth and 12th week {P = 0.078}), and also of the baseline values of the osteotomized right (R) femurs (P = 0.050) (pairwise comparisons indicate a difference between the sixth and 12th week {P = 0.046}).

There is a statistically significant difference between baseline and euthanasia values of the sixth week (p < 0.001), eighth week (P < 0.001), 10th week (P = 0.007), and 12th week (P < 0.001) for the total BMD of the control left (L) femurs, and only at six weeks (P = 0.007) for the total BMD of the osteotomized right (R) femurs.

MDF results

There is a statistically significant difference between the groups (six weeks vs eight weeks vs 10 weeks vs 12 weeks) of the euthanasia control left (L) femurs (P < 0.001) (pairwise differences of the eighth week between the sixth {P < 0.001}, 10th {P < 0.001}, and 12th week {P < 0.001}, respectively). There is also a statistically significant difference between MDF time measurements (six weeks vs eight weeks vs 10 weeks vs 12 weeks) of the baseline osteotomized right (R) femurs (P = 0.010) (pairwise differences of the eighth week between the 10th {P = 0.037} and 12th week {P = 0.037}, respectively, Table 1).

There is also a statistically significant difference between MDF euthanasia values of the osteotomized right (R) femurs (P < 0.001) (pairwise differences of the sixth week between the eighth {P < 0.001}, 10th {P < 0.001}, and 12th week {P < 0.001}, respectively). Finally, there is no statistically significant difference for the MDF baseline values of the control left (L) femurs (P = 0.102). There is a statistically significant difference between the baseline and euthanasia MDF values at the eighth week (P < 0.001) of the control left (L) femurs, and at the sixth (P < 0.001), eighth (P < 0.001), 10th (P = 0.004), and 12th week (P = 0.004) of the osteotomized right (R) femurs.

SSIp results

There is no statistically significant difference between baseline and euthanasia SSIp values of all groups of the left (L) control femur. However, there is a statistically significant difference between baseline and euthanasia evaluation at the sixth week (P = 0.039), eighth week (P = 0.004), and the 12th week (P = 0.032) of the SSIp value of the osteotomized right (R) femur (Table 1). It is noteworthy that there is no statistically significant difference in the 10th week (P = 0.420).

There is no statistically significant difference between groups (six weeks vs eight weeks vs 10 weeks vs 12 weeks) of the baseline SSIp values of the control left (L) femur (P = 0.491) and the osteotomized right (R) femur (P = 0.950), as well as of the euthanasia values of the control left (L) femur (P = 0.076) and the osteotomized right (R) femur (P = 0.108).

There is no statistically significant difference between baseline total BMD, MDF, and SSIp values of the control and osteotomized femur of all groups (data not shown). Comparisons between euthanasia total BMD, MDF, and SSIp values the control and osteotomized femur of each group can be seen in Table 2.

Total BMD results

There is no statistically significant difference between euthanasia total BMD values of the control and osteotomized femur of all groups. The BMD values of the osteotomized femur show a gradual increase throughout time, with values at 10-12 weeks having reached the values of the control femur at six weeks (Table 2).

MDF results

The MDF euthanasia values of the osteotomized femur compared to the control femur are statistically significantly higher for all groups, i.e., sixth week (P < 0.001), eighth week (P < 0.001), 10th week (P = 0.026), and marginally significant at the 12th week (P = 0.059). Their declining values throughout time, coming closer to control femur values, indicate an increase of bone quality (Table 2).

SSIp results

The euthanasia SSIp values of the osteotomized femur compared to the control femur are statistically significantly higher in the first three groups, i.e., sixth week (P = 0.010), eighth week (P = 0.014), and 10th week (P = 0.008), with no significant difference at the 12th week (P = 0.428, Table 2).

Correlations

There is a moderate negative correlation between MDF and total BMD at the sixth week (r = -0.424, P = 0.194) of the control femur. At the eighth week (r = -0.312, P = 0.323) and 10th week (r = -0.647, P = 0.043), respectively, there is a moderate and high negative correlation between MDF and total BMD for osteotomized femur. At the eighth week (r= -0.383, P = 0.219) and 10th week (r= -0.662, P = 0.037) respectively, there is a moderate and high negative correlation between MDF and total BMD for the ratio osteotomized/control femur (Table 3). At the sixth week (r = 0.473, P = 0.142), eighth week (r = 0.510, P = 0.090), and 10th week (r = 0.493, P = 0.147), there is a high positive correlation between MDF and SSIp of the left control femur. At the 10th week (r= -0.350, P = 0.321), there is a moderate negative but not statistically significant correlation between MDF and SSIp of the right osteotomized femur. At the 10th week (r= -0.573, P = 0.054), there is a high negative correlation between MDF and SSIp of the ratio osteotomized/control (R/L) femur (Table 4). These correlations are also presented graphically in Figures 5, 6.

## Discussion

Evaluation of bone healing following the fracture is of paramount importance in clinical practice in planning and advising patients’ return to their activities safely and at the appropriate time. The evaluation of the progress of callus formation usually depends on radiographic examination. Various other techniques such as quantitative CT, dual-energy X-ray absorptiometry (DEXA), ultrasound, and bone stiffness testing have been implemented [3]. However, none of these has been proven reliable for everyday clinical practice.

The present study aimed to investigate whether the MDF method is reliable in assessing and monitoring the process of bone fracture healing, compared to the established pQCT method. The surgical model used to create the fracture for this purpose was an accurate, standardized, and reproducible method in rats. It relies upon a specific osteotomy width and the insertion of a removable femoral intramedullary nail with two interlocking pins, proximal and distal to the osteotomy, thus providing a stable fixation by preventing rotation, securing the femoral axis, and therefore, promoting uneventful fracture healing [15,16].

The pQCT method was used to measure volumetric bone mineral density at the callus site from six to twelve postoperative weeks, and to investigate how its results correlate with the MDF results. It should be noted that pQCT facilities are not common in the health care community, therefore not widely accessible and of substantial cost for the healthcare systems. A more accessible and portable method to evaluate fracture healing among community-dwelling individuals during the rehabilitation period would be an attractive and beneficial alternative.

In this study, femoral fracture healing in rats was monitored up to 12 weeks postoperatively. According to the evidence in rats of this age group, 12 weeks is the earliest time point the fracture union can be achieved [17,18]. For the study's aim, we considered monitoring the significant changes in the mechanical properties of the callus that occur during the initial phases of fracture healing before the following prolonged and ongoing remodeling phase. It is noteworthy that in all parameters measured (total BMD, MDF, and SSIp) there was no statistically significant difference between osteotomized right and control left femurs in the 12-week group.

The pQCT results demonstrated that baseline total BMD values of the control (left) and osteotomized (right) femurs of all groups were similar, as expected. Additionally, postoperative total BMD values of the osteotomized right femurs were lower than those of the controls, with their values improving throughout time (Table 1). The higher euthanasia total BMD values of the control left femurs of all groups (Table 2) may be explained by the increased weight-bearing that they withstood *via* the animals' preference to load the control leg, increasing bone density. Utvåg and Reikerås have also reported an increase of all the mechanical variables, as well as bone mineral content and BMD, in the reference group of their study from six to 12 weeks [19]. It would also be reasonable to assume that during the observation period of the present study (ranging from one and a half to three months), BMD is expected to increase, similarly to the strength of intact rat femurs, which increases with age [20].

The MDF results demonstrated that baseline values of the control and osteotomized femurs were similar, as expected, as were the euthanasia MDF values of the control left femurs. An increase of MDF values of the osteotomized femurs postoperatively was observed in all groups following osteotomy, indicating the deteriorated bone quality due to surgery (Table 1). It should be noted that MDF values of the osteotomized femurs decreased from 6 to 10 weeks with the improvement of bone quality throughout time after osteotomy. During this period, the callus is strengthened continuously by the mineralization process. Later on, during the remodeling phase at 10-12 weeks, the initially formed so-called “woven bone” is resorbed, before it changes to mature lamellar bone [1-3]. This process explains to a certain degree the small increase of MDF values (slight deterioration of mechanical properties) observed in this late phase of our study. The rigidity of the nail used is certainly another contributing factor to this inverse trend observed in the MDF values at 12 weeks. ‘The RatNail system’ is an interlocked intramedullary nail made of medical grade stainless steel 1.4441 (316L) which can be held accountable for a stress shielding effect. Previous experimental studies on bone healing have shown that by applying a rigid type of fixation, callus strength is achieved at a higher rate in the initial phases of healing [21]. Although rigid fixation’s initial effect is beneficial, it results in cortical bone resorption and decreased callus strength especially during the remodeling phase, leading to mechanical weakening of the fractured bone [22,23]. This has also been seen in studies on intramedullary nailing of rats’ intact femora where when rigid nails were used, a higher degree of stress shielding and greater porosity was observed compared to more flexible nails [24,25].

Our results showed a high correlation between MDF and total BMD and a moderate correlation between MDF and SSIp, which supports the validity of MDF in assessing fracture healing. The same results were found for the ratio osteotomized/control femur. The highest correlations for the osteotomized right femurs between MDF and total BMD (r = -0.647, P = 0.043), and MDF and SSIp (r = -0.350, P = 0.321) were observed at 10 weeks. Low or moderate correlations were noted between the pQCT and MDF measurements at eight to 12 weeks post-osteotomy, as indicated in Tables 3, 4 and Figures 5, 6. It may be considered that measurements at six weeks are not indicated for estimation of the callus' mechanical properties, as it is premature, which is why at that time the correlations are not significant.

The improvement of bone quality (callus formation) of the right femur during the consecutive time points (six, eight, 10, and 12 weeks) can be seen in Figures 1, 3. One rat that did not develop callus formation at 6 weeks post-osteotomy despite the stable fixation (Figure 2) may be attributed to subclinical infection, as the non-union presented atrophic features. Clearly, postoperative MDF values are higher than preoperative baseline values, indicating bone quality deterioration due to osteotomy (Table 1). Taking into account the overlapping of the fracture healing phases, the biological process of remodeling is expected to occur around the 10th to 12th week, causing, inevitably, changes to the mechanical properties of the healing bone [1-3]. This progress is confirmed by the measurements noted by the established pQCT method. The MDF measurements are correlated with those of the pQCT method in a way that suggests that this method, mainly applied on metallic structures, could be used as a quantitative estimation of bone fracture healing. Further data in support of our present results are expected after the full analysis of this study with additional technical methods, including *ex vivo* femoral strength data.

Limitations

Ideally, the longitudinal monitoring of the same animals by both methods, pQCT, and MDF, throughout the study period, would potentially give more information on the fracture healing process evaluation, instead of having different animal groups for each time point. This may be considered a limitation of our study design. However, given the fact that all rats were purchased from the same commercial breeder, were of the same age, and littermates randomized into each group, it could be accepted that their differences would be negligible.

## Conclusions

In this experimental study, we aimed to investigate whether MDF can be used as a simple and reliable method for monitoring the process of bone fracture healing, compared to the established pQCT method. Based on our findings in this fracture healing animal model, the results from the MDF method are reliable and highly correlated with the total bone mineral density and moderately with the SSI polar values obtained by the pQCT method of bone quality measurement. As pQCT scanners are rarely available in secondary or even tertiary care hospital settings of large cities, the MDF method may be an attractive portable alternative to monitor fracture healing in the community and provide more detailed and quantitative information for the appropriate initiation of weight-bearing in fracture patients. This method could also be applied to monitor patients with osteoporosis or cases with imminent risk of fracture. Further experimental studies with intermediate time point measurements, as well as clinical studies, may yield additional information.
